# Supplementary material for: Rift Valley fever in northern Senegal: A modelling approach to analyse the processes underlying virus circulation recurrence
Source: PLoS Negl Trop Dis. 2020 Jun 1;14(6):e0008009. doi: 10.1371/journal.pntd.0008009 (PMC7289439; doi:10.1371/journal.pntd.0008009)
Supplement: S1 Table — (DOCX) [file pntd.0008009.s002.docx]

**S1. CHARACTERISTICS OF THE NOMADIC HERDS SURVEYED**

Most sampled herds included cattle, goats, sheep and donkeys. Sheep was the dominant species in most herds, although cattle represented >50% of animals in two herds (herds 4 and 22). The distribution of the size of the 22 herds is provided in Figure S2

**Table S1. Composition of the sampled herds**

| Herd ID | Session | Herd type | Total size | Number of bovines (%) | Number of ovines (%) | Number of caprines (%) | Number of donkeys (%) |
| --- | --- | --- | --- | --- | --- | --- | --- |
| 1 | 1 | L^a^ | 267 | 15 (5,6) | 150 (56,2) | 100 (37,4) | 2 (0,75) |
| 2 | 1 | S^b^ | 303 | 80 (26,4) | 160 (52,8 | 50 (16,5) | 13 (4,3) |
| 3 | 1 | L | 237 | 70 (29,5) | 100 (42,2) | 34 (14,3) | 33 (13,9) |
| 4 | 1 | S | 272 | 150 (55,1) | 47 (17,3) | 47 (17,3) | 28 (10,3) |
| 5 | 2 | L | 136 | 30 (22) | 80 (58,8) | 20 (14,7) | 6 (4,4) |
| 6 | 2 | L | 388 | 25 (6,4) | 250 (64,4) | 100 (25,8) | 13 (3,4) |
| 7 | 2 | S | 403 | 80 (19,9) | 200 (49,6) | 100 (24,8) | 23 (5,7) |
| 8 | 2 | S | 169 | 80 (47,3) | 40 (23,7) | 40 (23,7) | 9 (5,3) |
| 9 | 3 | L | 1550 | 400 (25,8) | 1000 (64,5) | 100 (6,5) | 50 (3,2) |
| 10 | 3 | L | 437 | 38 (8,7) | 300 (68,6) | 90 (20,6) | 9 (2,1) |
| 11 | 3 | S | 107 | 7 (6,5) | 43 (40,2) | 50 (46,7) | 7 (6,5) |
| 12 | 3 | L | 158 | 60 (38) | 80 (23,7) | 10 (6,3) | 8 (5,1) |
| 13 | 3 | S | 205 | 15 (7,3) | 130 (63,4) | 40 (19,5) | 20 (9,8) |
| 14 | 4 | L | 307 | 6 (2) | 210 (68,4) | 80 (26,1) | 11 (3,6) |
| 15 | 4 | L | 102 | 0 (0) | 80 (78,4) | 13 (12,7) | 9 (8,8) |
| 16 | 4 | S | 336 | 80 (23,8) | 200 (59,2) | 40 (11,9) | 16 (4,8) |
| 17 | 4 | L | 193 | 60 (31,1) | 50 (25,9) | 60 (31,1) | 23 (11,9) |
| 18 | 4 | S | 85 | 8 (9,4) | 40 (47,1) | 30 (35,3) | 7 (8,2) |
| 19 | 5 | S | 1716 | 300 (17,5) | 1200 (69,9) | 160 (9,3) | 56 (3,3) |
| 20 | 5 | S | 340 | 0 (0) | 300 (88,2) | 25 (7,4) | 15 (4,4) |
| 21 | 5 | L | 93 | 20 (21,5) | 30 (32,3) | 40 (43) | 3 (3,2) |
| 22 | 5 | S | 96 | 50 (52,1) | 15 (15,6) | 20 (20,8) | 11 (11,5) |

^a^ Long-range nomads. ^b^ Short-range nomads.
