# Supplementary figures and images for: Rift Valley fever in northern Senegal: A modelling approach to analyse the processes underlying virus circulation recurrence
Source: PLoS Negl Trop Dis. 2020 Jun 1;14(6):e0008009. doi: 10.1371/journal.pntd.0008009 (PMC7289439; doi:10.1371/journal.pntd.0008009)

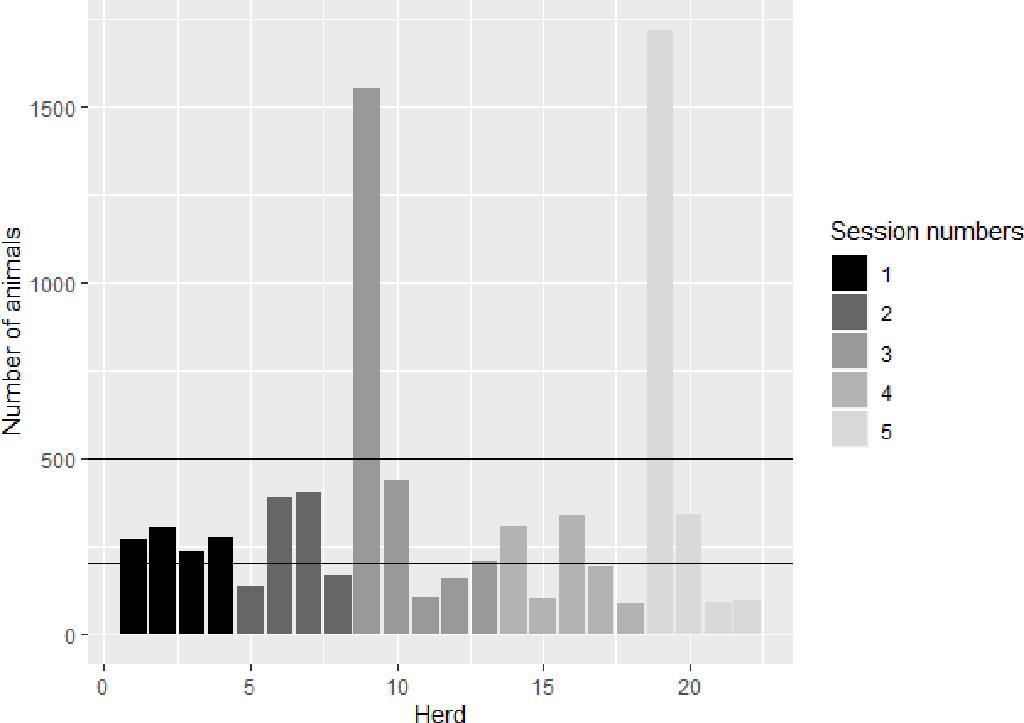

Supplement: S1 Fig — (TIF) [file pntd.0008009.s003.tif]
