# Supplementary material for: Rift Valley fever in northern Senegal: A modelling approach to analyse the processes underlying virus circulation recurrence
Source: PLoS Negl Trop Dis. 2020 Jun 1;14(6):e0008009. doi: 10.1371/journal.pntd.0008009 (PMC7289439; doi:10.1371/journal.pntd.0008009)
Supplement: S1 Text — (DOCX) [file pntd.0008009.s004.docx]

**S1 Text. EPIDEMIOLOGICAL MODEL**

The model was a compartmental deterministic model operating in discrete time with a daily time step. The model compartments represented the health states of vectors and hosts (Fig. 2). Vector health states were *S* (susceptible vectors), *L* (infected bot non-infectious vectors -during the extrinsic incubation period), and *I* (infectious vectors that may transmit RVFV to the susceptible hosts upon which they feed). Host health states were *S* (susceptible animals), *I* (viremic animals, which may transmit RVFV to vectors feeding upon them), and *R* (immune animals). Host populations were further stratified by age (with yearly age classes) and by sex/physiological status, with three different categories (males, empty females and gestating females). At a given time step *t*, the state of vector populations $v\in V=\left\{ Ae, Cx \right\}$ was described by the proportion of females in each health state: $S_{v}(t)$, $L_{v}(t)$ and $I_{v}(t)$ (with $S_{v}\left( t \right)+L_{v}\left( t \right)+I_{v}\left( t \right)=1$), and by $G_{v}(t)$: the proportion of infected eggs. The state of host populations of species *h* (cattle or small ruminant) belonging to breeders of group *m* (sedentary or nomadic) was described by the number of animals in each health state, structured by age *a* and by sex/physiological status *q*: $S_{h}^{m,a,q}(t)$, $I_{h}^{m,a,q}(t)$ and $R_{h}^{m,a,q}(t)$. Model dynamics was made of three distinct processes sequentially simulated at each time step: the population dynamics (birth and death of vectors and hosts), the infection dynamics (RVFV transmission) and the population renewal (arrival and departure of nomadic breeders).

**Population dynamics in vectors and hosts**

The population dynamics of vectors was not explicitly represented; we used instead the outputs of the entomological model (EM) elaborated by Tran et al.[[36](#_ENREF_36)] in the same study area, computed with a daily time step (Fig. 2). This model reproduced the demographic dynamics of *Aedes vexans* and *Culex poicilipes* mosquitoes around a temporary pond, based on the evolution of pond surface, rainfall, temperature and humidity [[42-46](#_ENREF_42)]. Based on the outputs of the EM, for each vector population $v$, at time step *t*, the proportion of adult females in each of the three health states after mortality and emergence was denoted $S_{v}^{*}\left( t \right)$, $L_{v}^{*}\left( t \right)$, $I_{v}^{*}\left( t \right)$, and computed using equations (1)-(3).

1. $S_{v}\left( t^{*} \right)=S_{v}\left( t \right) \sigma_{v}\left( t \right)+\left( 1-G_{v}\left( t \right) \right) \left( 1-\sigma_{v}\left( t \right) \right)$
2. $L_{v}\left( t^{*} \right)=L_{v}\left( t \right) \sigma_{v}\left( t \right)$
3. $I_{v}\left( t^{*} \right)=I_{v}\left( t \right) \sigma_{v}\left( t \right)+G_{v}\left( t \right) \left( 1-\sigma_{v}\left( t \right) \right)$

Where $\sigma_{v}\left( t \right)$ is the daily survival rate of adult females for time step *t*: $\sigma_{v}\left( t \right)=\frac{N_{v}\left( t \right)-n_{v}\left( t \right)}{N_{v}\left( t+1 \right)}$, computed from the EM outputs $N_{v}\left( t \right)$ the number of adult females at the beginning of time step *t*, and $n_{v}\left( t \right)$ the number of these females that die during the time step.

**Table A. Parameters of mosquito population dynamics parameterized by outputs of the entomological model (EM [1])**

| Notation | Description | Value |
| --- | --- | --- |
| $N_{v}\left( t \right)$ | Number of adult female mosquitoes at the beginning of time step *t* | EM |
| $n_{v}\left( t \right)$ | Number of adult female mosquitoes dying during time step *t* | EM |
| $M_{v}\left( t \right)$ | Proportion of adult female mosquitoes of population$v$ that take a blood meal during time step *t* | EM |
| $\theta(t)$ | Temperature et time step t | EM |
| $O_{v}\left( t \right)$ | Proportion of female mosquitoes of population $v$ which lay down eggs during the time step t | EM |
| $E_{v}\left( t \right)$ | Number of eggs of population $v$ in the epidemiological system at the beginning of time step *t* | EM |
| $e_{v}\left( t \right)$ | Number of eggs laid down during time step t by females of population $v$ | EM |

The state of host populations after inseminations, births and culls was denoted by $S_{h}^{m,a,q}\left( t^{*} \right)$, $I_{h}^{m,a,q}\left( t^{*} \right)$, and $R_{h}^{m,a,q}\left( t^{*} \right)$ for animals in health states *S*, *I* and *R*, respectively. It was modelled by equations (4)-(6):

1. $S_{h}^{m,1,q}\left( t^{*} \right)=S_{h}^{m,1,q}\left( t \right)\left( 1-\mu_{h}^{m,1}(t) \right)+\frac{1}{2}\rho_{h}^{m} \sum_{a\in A} B_{h}^{m,a}(t)$

for each $q\in Q=\left\{ M, EF \right\}$ (*M*: male, *EF*: empty female)

1. $X_{h}^{m,a,EF}\left( t^{*} \right)= X_{h}^{m,a,EF}\left( t \right)\left( 1-\mu_{h}^{m,a}(t) \right)+B_{h}^{m,a}\left( t \right)-f_{h}^{m}\left( t \right) X_{h}^{m,a,EF}$

for each $X\in\left\{ S,I,R \right\}$

1. $X_{h}^{m,a,GF}\left( t^{*} \right)= X_{h}^{m,a,GF}\left( t \right)\left( 1-\mu_{h}^{m,a}(t) \right)-B_{h}^{m,a}\left( t \right)+f_{h}^{m}\left( t \right) X_{h}^{m,a,EF}$

for each $X\in\left\{ S,I,R \right\}$

where:

- $\mu_{h}^{m,a}(t)$ is the culling rate of animals of species *h*, age *a*, that belong to a breeder of group *m*;
- $\rho_{h}^{m}$ denotes the average prolificity (number of viable products per calving/lambing) of females of species *h* belonging to breeders of group *m*;
- $B_{h}^{m,a}(t)$ denotes the number of these females which calve/lamb at time step t: $B_{h}^{m,a}\left( t \right)=b_{h}^{m}\left( t \right) \sum_{X\in\left\{ S,I,R \right\}} X_{h}^{m,a,GF}$, with the $b_{h}^{m}\left( t \right)$ and $f_{h}^{m}\left( t \right)$ representing respectively the proportion of pregnant females calving/lambing and the proportion of non-gestating females becoming pregnant.

**Table B. Parameters of host population dynamics**

| Notation | Description | Value | Source |
| --- | --- | --- | --- |
| $\mu_{h}^{m,a}(t)$ | Culling rate of animals of species *h*, age *a*, that belong to a breeder of group *m* | Females: calibrated to obtain a stable yearly cycle of the number of females  Males: calibrated to obtain a stable yearly cycle of the number of males, with a proportion of 15% of males in the population | F. Sow, pers. comm. |
| $b_{h}^{m}\left( t \right)$ | Proportion of calving or lambing occurring at the calendar day corresponding to time step *t* in the yearly production cycle of animals of species *h* belonging to breeders of group *m* | Cattle: U(01/06-31/08)  Small Ruminants, sedentary breeders: U(01/09-31/12)  Small Ruminants, nomadic breeders: U(01/08-31/10)  (U: uniform distribution) | F. Sow, pers. comm. |
| $\rho_{h}^{m}$ | Average prolificity (number of viable products per calving/lambing) of animals of species *h* belonging to breeders of group *m* | $\rho_{Ca}^{Sd}=\rho_{Ca}^{Nd}=0.82$  $\rho_{Sr}^{Sd}=\rho_{Sr}^{Nd}=1.5$ | F. Sow, pers. comm. |
| $f_{h}^{m}\left( t \right)$ | Proportion of females becoming pregnant at the calendar day corresponding to time step *t* in the yearly production cycle of animals of species *h* belonging to breeders of group *m* | Cattle: U(01/03-30/1) for 50% of animals  Small Ruminants, sedentary breeders: U(01/04-31/07)  Small Ruminants, nomadic breeders: U(01/03-31/05)  (U: uniform distribution) | F. Sow, pers. comm. |

**Infection dynamics in vectors and hosts**

Vectors could get infected by RVFV either when feeding on a viraemic host, or vertically (transmission from an infected female to its eggs). In vectors, the transmission dynamics was modelled by equations (7)-(10):

1. $S_{v}\left( t+1 \right)=S_{v}\left( t^{*} \right) \left( 1-\lambda_{v}\left( t \right) \right)$
2. $L_{v}\left( t+1 \right)=L_{v}\left( t^{*} \right)\left( 1-\frac{1}{\varphi_{v}(t)} \right)+S_{v}\left( t^{*} \right) \lambda_{v}\left( t \right)$
3. $I_{v}\left( t+1 \right)=I_{v}\left( t^{*} \right)+L_{v}\left( t^{*} \right)\frac{1}{\varphi_{v}(t)}$
4. $G_{v}\left( t+1 \right)=I_{v}\left( t^{*} \right) \omega_{v} \left( 1-r_{v}\left( t \right) \right)+G_{v}\left( t \right) r_{v}\left( t \right)$

where $\varphi_{v}(t)$ denoted the temperature-dependent duration of extrinsic incubation, $\lambda_{v}\left( t \right)$ the force of infection (FOI) applied on susceptible females, $\omega_{v}$ the probability that an infected female transmits the virus to its eggs, and $r_{v}\left( t \right)$ the fraction of eggs present at *t*+1 that were already present at *t* (Table C).

**Table C. Parameters of transmission dynamics in mosquito populations**

| Notation | Description | Value | Source |
| --- | --- | --- | --- |
| $\varphi_{v}(t)$ | Temperature-dependent duration of extrinsic incubation at time-step *t* | $\varphi_{Ae}\left( t \right)=18.9-0.3 \theta(t)$  $\varphi_{Cx}\left( t \right)=11.3-0.3 \theta(t)$ | [[2](#_ENREF_47)] |
| $\pi_{h,v}$ | Trophic preference of mosquitoes of population $v$ for hosts of species *h* | $\pi_{Ca,Ae}=1$  $\pi_{Sr,Ae}=0.3$  $\pi_{Ca,Cx}=0.2$  $\pi_{Sr,Cx}=0.2$ | [[2](#_ENREF_47)] |
| $\gamma_{h,v}$ | Probability for female mosquitoes of population $v$ to get infected by RVFV when biting a viraemic host of species *h* | $\gamma_{Ca,Ae}=\gamma_{Sr,Ae}=0.38$  $\gamma_{Sr,Ae}=\gamma_{Ca,Cx}=0.20$ | [2] |
| $\omega_{v}$ | Probability that an infected female mosquito (*I* state) of population $v$ transmits the virus to its eggs |  | Estimated |
| $r_{v}\left( t \right)$ | Fraction of eggs present at *t* that were already present at *t*-1 | Time-varying | EM |
| $M_{v}\left( t \right)$ | proportion of adult females of population$v$ taking a blood meal during time step *t* | Time-varying | EM |

*EM: Entomological Model [1]

The FOI applied on susceptible vectors was:

1. $\lambda_{v}\left( t \right)=\sum_{h\epsilon H} M_{v}\left( t \right)\frac{\pi_{h,v} N_{h}(t)}{\sum_{k\epsilon H} \pi_{k,v} N_{k}(t)} \gamma_{h,v}\frac{I_{h}^{tot}(t)}{N_{h}(t)}$

where $\gamma_{h,v}$ denoted the probability for a female mosquito to get infected when biting a viraemic host, and $\pi_{h,v}$ was the trophic preference of mosquitoes of population $v$ for hosts of species *h.* The term $\frac{\pi_{h,v} N_{h}(t)}{\sum_{k\epsilon H} \pi_{k,v} N_{k}(t)}$ thus denoted the probability that an adult female, when taking a blood meal, bites a host of species *h* (with, for species *h*, $I_{h}^{tot}(t)$ the total number of viraemic hosts, and $N_{h}(t)$ the total number of hosts). $M_{v}\left( t \right)$, the daily proportion of females taking a blood meal, was an output of EM (Table A, [[1](#_ENREF_48)]).

**Table D. Parameters of transmission dynamics in host populations**

| Notation | Description | Value | Source |
| --- | --- | --- | --- |
| $\eta_{h}^{a}$ | daily mortality rate of viraemic animals of age *a*, due to RVFV infection, computed from the cumulated mortality rate for the whole viraemia period | ${1-\left( {1-\eta}_{Sr}^{1} \right)}^{\alpha_{Sr}}=0.7$  ${1-\left( {1-\eta}_{Sr}^{>1} \right)}^{\alpha_{Sr}}=0.1$  $\eta_{Ct}^{1}=\eta_{Sr}^{>1}=0$ | [[2](#_ENREF_49)] |
| $\alpha_{h}$ | Duration of viraemia for hosts of species *h* | $\alpha_{Ct}=\alpha_{Sr}=5$ days | [[2](#_ENREF_49)] |
| $\beta$ | Transmission parameter to hosts exposed to abortion products or to calving/lambing products from viraemic animals |  | Estimated |
| $\psi$ | Scaling factor for vector population sizes |  | Estimated |
| $\gamma_{v,h}$ | Probability for a host of species *h* to get infected by RVFV when bitten by an infectious female mosquito of population $v$ | $\gamma_{Ae,Sr}=\gamma_{Ae,Ct}=0.70$  $\gamma_{Cx,Sr}=\gamma_{Cx,Ct}=0.78$ | [[2](#_ENREF_47)] |

Two between-host transmission routes were considered: vector-borne and direct, when viraemic hosts abort of calve/lamb (Fig. 2), thus exposing susceptible animals to highly infectious materials. A disease-induced mortality was also taken into account. The state of host populations after RVFV infections, immunizations, abortions and deaths due to the disease was denoted by $S_{h}^{m,a,q}\left( t^{**} \right)$, $I_{h}^{m,a,q}\left( t^{**} \right)$, and $R_{h}^{m,a,q}\left( t^{**} \right)$ for animals in health states *S*, *I* and *R*, respectively. It was modelled by equations (12)-(14):

1. $S_{h}^{m,a,g}\left( t^{**} \right)=S_{h}^{m,a,g}\left( t^{*} \right) \left( 1-\lambda_{h}(t) \right)$
2. $I_{h}^{m,a,g}\left( t^{**} \right)=I_{h}^{m,a,g}\left( t^{*} \right) (1-\eta_{h}^{a}) \left( 1-\frac{1}{\alpha_{h}} \right)+S_{h}^{m,a,g}\left( t^{*} \right) \lambda_{h}(t)$
3. $R_{h}^{m,a,g}\left( t^{**} \right)=R_{h}^{m,a,g}\left( t^{*} \right)+ I_{h}^{m,a,g}\left( t^{*} \right) (1-\eta_{h})\frac{1}{\alpha_{h}}$

where $\eta_{h}^{a}$ denoted the daily mortality rate of viremic animals due to RVFV infection, $\alpha_{h}$ was the duration of viraemia, and $\lambda_{h}(t)$ the FOI exerted on hosts.

This FOI was the sum of three components, the FOI due to exposure to abortion products $\lambda_{h}^{A}\left( t \right)$, the FOI due to exposure to calving or lambing products of viraemic females $\lambda_{h}^{C}\left( t \right)$, and the FOI exerted by vectors: $\lambda_{h}^{V}\left( t \right)$ (equations (15)-(18)).

1. $\lambda_{h}\left( t \right)=\lambda_{h}^{A}\left( t \right)+\lambda_{h}^{C}\left( t \right)+\lambda_{h}^{V}\left( t \right)$
2. $\lambda_{h}^{A}\left( t \right)=\beta\chi_{h} \sum_{k\epsilon H,a\epsilon A,m\in M} I_{h}^{m,a,GF}\left( t^{*} \right)$
3. $\lambda_{h}^{C}\left( t \right)=\beta\sum_{k\epsilon H,a\epsilon A,m\in M} b_{h}^{m}\left( t \right)I_{h}^{m,a,g}\left( t^{*} \right)$
4. $\lambda_{h}^{V}\left( t \right)=\sum_{v\epsilon V} M_{v}\left( t \right)\frac{\pi_{h,v} N_{h}(t^{*})}{\sum_{k\epsilon H} \pi_{k,v} N_{k}(t^{*})}\frac{\psi N_{v}(t)}{N_{h}(t^{*})} \gamma_{v,h} I_{v}(t)$

Where $\beta$ was the transmission parameter by exposure to infectious materials, $\chi_{h}$ was the daily abortion rate of viraemic females, and $\gamma_{v,h}$ denoted the probability for a host of species *h* to get infected by RVFV when bitten by an infectious female mosquito of population $v$. Two supplementary parameters were outputs of EM [[1](#_ENREF_48)]: $b_{h}^{m}\left( t \right)$ the proportion of females that calve/lamb at time *t*, and $M_{v}\left( t \right)$ the proportion of adult females of population$v$ that take a blood meal during time step *t*. Because vector population dynamics resulted from an independent entomological model, a scaling factor $\psi$ was introduced for the vector population size: the term $\frac{\psi N_{v}(t)}{N_{h}(t^{*})}$ (with $N_{v}(t)$ an output of EM denoting the number of females) was thus the vector-to-host ratio.

**Population renewal of hosts**

Ruminant sedentary populations (denoted *Sd*) were assumed closed, without any external renewal:

1. $X_{h}^{Sed,a,g}\left( t+1 \right)=X_{h}^{Sed,a,g}\left( t^{**} \right)$ *for each* $X\in\left\{ S,I,R \right\}$

Oppositely, nomadic populations (denoted *Nd*) were considered open, with daily arrival of some breeders and departure of others , the number of breeders being higher and the duration of their stays longer during the rainy season [[3](#_ENREF_20)]. The dynamic of the renewal of nomadic populations was modelled according to these hypotheses by equations (20)-(22).

1. $S_{h}^{Nd,a,g}\left( t+1 \right)=S_{h}^{Nd,a,g}\left( t^{**} \right) \left( 1-\tau\left( t \right) \right)+1_{\Delta_{h}\left( t \right)>0}\left( 1-\theta\right) \Delta_{h}\left( t \right) \Gamma_{h}^{a}+\left( 1-1_{\Delta_{h}\left( t \right)>0} \right) \Delta_{h}\left( t \right)\frac{S_{h}^{Nd,a,g}\left( t^{**} \right)}{N_{h}^{Nd}\left( t^{**} \right)}$
2. $I_{h}^{Nd,a,g}\left( t+1 \right)=I_{h}^{Nd,a,g}\left( t^{**} \right) \left( 1-\tau\left( t \right) \right)+\left( 1-1_{\Delta_{h}\left( t \right)>0} \right) \Delta_{h}\left( t \right)\frac{I_{h}^{Nd,a,g}\left( t^{**} \right)}{N_{h}^{Nd}\left( t^{**} \right)}$
3. $R_{h}^{Nd,a,g}\left( t+1 \right)=R_{h}^{Nd,a,g}\left( t^{**} \right) \left( 1-\tau\left( t \right) \right)+1_{\Delta_{h}\left( t \right)>0}\theta\Delta_{h}\left( t \right) \Gamma_{h}^{a}+\left( 1-1_{\Delta_{h}^{m}\left( t \right)>0} \right) \Delta_{h}\left( t \right)\frac{R_{h}^{Nd,a,g}\left( t^{**} \right)}{N_{h}^{Nd}\left( t^{**} \right)}$

where:

- $\tau\left( t \right)$ is the renewal rate for species *h* at time step *t*: this rate was assumed to follow a trapezoidal yearly dynamic, with

(i) a stable high value (i.e. shorter stays) during the dry season, denoted $\tau_{ds}$,

(ii) a linear decrease during the 15 days before the beginning of the rainy season denoted $T_{y}^{beg}$ (where y is the year of time step t),

(iii) a stable low value during the rainy season, denoted $\tau_{rs}$, followed by a

(iv) linear increase during the 15 days after the end of the rainy season denoted $T_{y}^{end}$, returning back to the high level of the dry season.

- $\theta$ is the proportion of immune animals among those which enter the study area,
- $\Delta_{h}\left( t \right)$ is the net change of population size during time step (i.e. a number of animals, positive if population is increasing, null if it is stable and negative if the population is decreasing): $\Delta_{h}\left( t \right)=\bar{N_{h}}\left( t+1 \right)- N_{h}^{Nd}\left( t^{**} \right)$, with $\bar{N_{h}}\left( t \right)$ the expected number of hosts of species *h* at time step t belonging to nomadic breeders, and $N_{h}^{Nd}\left( t \right)$ the total number of these animals in the epidemiological system. Similarly to the renewal rate, $\bar{N_{h}}\left( t \right)$ was assumed to follow a trapezoidal yearly dynamic, with

(i) a stable low value during the dry season, denoted $\bar{N_{h}^{ds}}$,

(ii) a linear increase during the 15 days before the beginning of the rainy season,

(iii) a stable low value during the rainy season, denoted $\bar{N_{h}^{rs}}$, followed by a

(iv) a linear decrease during the 15 days after the end of the rainy season, returning back to the low level of the dry season.

- $\Gamma_{h}^{a}$ is the proportion of animals of age *a* among those of species *h*, according to the age structure of the herds/flocks kept by nomadic breeders, considered constant for a simplification purpose. This age structure was computed from the initial state of nomadic populations.

**Table E. Parameters of host population renewal, resulting from the epidemiological study conducted in the study area**

| Notation | Description | Value | Source |
| --- | --- | --- | --- |
| $\tau_{ds}$ | Daily renewal rate of hosts belonging to nomadic breeders, during the dry season | $1/\tau_{ds}=9$ days | Field survey |
| $\tau_{rs}$ | Daily renewal rate of hosts belonging to nomadic breeders, during the rainy season | $1/\tau_{rs}=16$ days | Field survey |
| $\theta$ | Proportion of immune animals among small ruminants entering the study area | Small ruminants: 0.12  Cattle: 0.14 | Field survey |
| $\bar{N_{h}^{ds}}$ | Expected number of hosts of species *h* kept by nomadic breeders during the dry season | $\bar{N_{Ct}^{ds}}=6$ animals  $\bar{N_{Sr}^{ds}}=29$ animals | Field survey |
| $\bar{N_{h}^{rs}}$ | Expected number of hosts of species *h* kept by nomadic breeders during the rainy season | $\bar{N_{Ct}^{rs}}=382$ animals  $\bar{N_{Sr}^{rs}}=1273$ animals | Field survey |

We estimated the parameters of host population renewal, *i.e.* $\tau_{ds}$ and $\tau_{rs}$, as well as the proportion of immune animals among those entering the survey area ($\theta)$ from the results of the epidemiological survey conducted in nomadic breeders (see results section in main text). The expected numbers of hosts kept by nomadic breeders in the modelled epidemiological system during the rainy season ($\bar{N_{h}^{rs}}$) were estimated based on the results of the field survey (Table 6) conducted in nomadic herds. Assuming a homogeneous repartition of nomadic settlements in the study area, we used the results of the field survey conducted in the whole 86 sq. km study area (Table 6) to compute the average animal densities (number of cattle and small ruminants per sq. km) during the rainy season. The expected numbers of nomadic animals in the epidemiological system (i.e. 2.5 km from a given pond, see main text) were then calculated from these animal densities: 382 cattle and 1273 small ruminants (995 sheep and 278 goats). Concerning the dry season, the expected numbers of hosts kept by nomadic breeders was derived from data collected at the Younoufere drilling water place (see main text for details).

***Parameterization***

The yearly beginning and end dates of the rainy season were based on the outputs of the entomological model [1]. Each year, the beginning of the rainy season was set to the 1^st^ day at which the number of *Aedes* females exceeded one third of its maximal value in the yearly population dynamic. Similarly, the end of the rainy season was set to the last day at which the number of *Culex* females exceeded one third of its maximal value throughout the year (Table F).

**Table F. Dates of beginning and end of rainy seasons, computed each year from the abundance of *Aedes* and *Culex* females predicted by the entomological model.**

| Year | Beginning of the rainy season | End of the rainy season |
| --- | --- | --- |
| 2008 | 2008-08-26 | 2008-10-21 |
| 2009 | 2009-08-04 | 2009-10-22 |
| 2010 | 2010-07-13 | 2010-10-23 |
| 2011 | 2011-08-23 | 2011-10-28 |
| 2012 | 2012-07-28 | 2012-11-10 |
| 2013 | 2013-07-13 | 2013-10-29 |
| 2014 | 2014-07-07 | 2014-12-25 |
| 2015 | 2015-08-06 | 2015-12-29 |

**References**

1. Tran A, Fall A, Biteye B, Ciss M, Gimonneau G, et al. (2019) Spatial modelling of mosquito vectors for Rift Valley fever virus in Northern Senegal: integrating satellite-derived meteorological estimates in population dynamics models. Remote Sensing 11: 1024.

2. Fischer E, Boender G, Nodelijk G, de Koeijer A, van Roermund H. The transmission potential of Rift Valley fever virus among livestock in the Netherlands: a modelling study. Vet Res. 2013;44(58). doi: doi: 10.1186/1297-9716-44-58.

3. Belkhiria J, Lo M, Sow F, Martínez-López B, Chevalier V (2019) Application of Exponential Random Graph Models to determine Nomadic herders’ movements in Senegal. Trans Emerg Dis.
